# Utility of a Combined Diagnostic and Severity Scoring System Based on Complete Blood Count and Derived Immune‐Inflammatory Indicators in Children With *Mycoplasma pneumoniae* Pneumonia

**DOI:** 10.1002/jcla.70263

**Published:** 2026-06-06

**Authors:** Quan Liu, Li Wei, Liang Wang, Xing‐Mei Zhu, Hai‐Feng Wang, Ling Ge

**Affiliations:** ^1^ Department of Medical Laboratory Huaibei People's Hospital (Huaibei People's Hospital Attached to Bengbu Medical University) Huaibei People's Republic of China

**Keywords:** complete blood count, diagnostic value, immune‐inflammatory indicators, logistic regression analysis, *Mycoplasma pneumoniae*, ROC curve

## Abstract

**Background:**

This study assessed the diagnostic value of complete blood count (CBC) and derived immune‐inflammatory indicators for *Mycoplasma pneumoniae* pneumonia (MPP) in children and developed a practical scoring system for early diagnosis.

**Methods:**

A total of 1662 MP‐infected children were divided into pneumonia (*n* = 844) and non‐pneumonia (*n* = 818) groups. CBC parameters, derived immune‐inflammatory indicators and C‐reactive protein (CRP) were compared. Diagnostic performance was assessed using ROC curve analysis, and diagnostic models were constructed using multivariate logistic regression. Age‐stratified analysis addressed confounding. Model stability was evaluated via repeated stratified 10‐fold cross‐validation, calibration curves with Brier score, and decision curve analysis (DCA). A simplified scoring system was established and validated for clinical application.

**Results:**

Children in the pneumonia group were significantly younger (mean age 5.78 ± 3.41 years vs. 8.62 ± 4.92 years, *p* < 0.001). Lymphocyte count (LYM), lymphocyte percentage (LYM%), platelet count (PLT), plateletcrit (PCT), and the product of absolute lymphocyte count and platelet count (PLT × LYM) in the pneumonia group were significantly higher than in the non‐pneumonia group. Age‐stratified analysis confirmed these differences across all age subgroups (all *p* < 0.001). PLT × LYM, LYM%, PLT, and PCT were independent predictors. Internal validation showed a mean AUC of 0.7824 ± 0.0353, good calibration (Brier score = 0.1918 ± 0.0164), and positive net benefit on DCA. The scoring system achieved diagnostic rates of 71.5%, 51.2%, and 29.6% for high‐, medium‐, and low‐risk groups, respectively.

**Conclusion:**

MPP predominantly affects children under 7 years old. The simple scoring system, supported by robust internal validation, effectively stratifies pneumonia risk and offers a practical early diagnostic tool for primary care.

## Graphical abstract

This study developed a simple, low‐cost scoring system using routine complete blood count (CBC) and derived immune‐inflammatory indicators (LYM%, PLT, PCT, and PLT × LYM) for early diagnosis of *Mycoplasma pneumoniae* pneumonia (MPP) in 1662 children. Age‐stratified analysis confirmed that key indicators remained significantly higher in the pneumonia group across all age subgroups (all *p* < 0.001), with children under 7 years comprising 74.5% of cases. Internal validation via repeated 10‐fold cross‐validation demonstrated good discriminative ability (mean AUC 0.7824), calibration (Brier score 0.1918), and positive net benefit on decision curve analysis (DCA). The scoring system achieved diagnostic rates of 71.5%, 51.2%, and 29.6% for high‐ (≥ 6 points), medium‐ (3–5 points), and low‐risk (≤ 2 points) groups, respectively. This practical tool enables rapid risk stratification without specialized equipment, making it particularly suitable for primary healthcare settings.

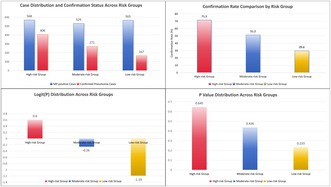

## 1 Introduction

*Mycoplasma pneumoniae* (MP) is a common pathogen causing community‐acquired pneumonia in children, accounting for 10% to 40% of such cases in individuals under 18 years old, with particularly high prevalence among preschoolers and adolescents [1]. MP infections exhibit cyclical epidemics, with peaks occurring every 3–7 years. Since 2023, MP outbreaks have emerged in multiple regions globally, and southern China has seen a sudden rise in positive detection rates since June [2]. Clinical manifestations of childhood MP infection are diverse, with early symptoms resembling common respiratory infections, leading to frequent misdiagnosis or delayed treatment. Some children may develop complications such as pulmonary consolidation and pleural effusion, posing a serious threat to pediatric health [3].

Current MP infection diagnosis relies on pathogen detection methods, including nucleic acid amplification and serological antibody testing, but these face limitations such as lengthy testing cycles, high costs, and potential false negatives in early stages [4]. Complete blood count (CBC), as a routine clinical test, offers the advantages of speed, convenience, and low cost. Its derived immune‐inflammatory indicators, such as lymphocyte‐platelet ratio (LYM × PLT), platelet‐lymphocyte ratio (PLR), and neutrophil‐lymphocyte ratio (NLR), have been demonstrated to reflect systemic inflammatory responses and immune status, holding significant value in infectious disease diagnosis [5, 6]. Research indicates that children with *Mycoplasma pneumoniae* pneumonia (MPP), particularly in the early stages of infection, exhibit heightened immune‐inflammatory responses characterized by enhanced platelet activation and lymphocyte function. These changes correlate closely with clinical disease severity [7].

This study compared the CBC and derived immune‐inflammatory indicators between children with MP infection complicated by pneumonia and those without pneumonia to identify core diagnostic indicators. A quantitative scoring system was developed using logistic regression models, and its stability and clinical utility were further evaluated using repeated cross‐validation, calibration analysis, and decision curve analysis (DCA). This study aims to provide a scientifically sound and clinically practical tool for rapid early diagnosis of MP‐associated pneumonia in children, thereby supporting early identification, disease assessment, and treatment decisions to reduce severe disease incidence.

## 2 Materials and Methods

### 2.1 Subjects

#### 2.1.1 Study Population and Selection Process

This study retrospectively included pediatric patients who visited our hospital between January 2023 and December 2025.

#### 2.1.2 Initial Screening

Children with laboratory‐confirmed MP infection (positive MP‐DNA and/or MP‐IgM) were initially identified. All included patients were newly diagnosed, untreated, and without documented underlying diseases (such as congenital heart disease or immunodeficiency disorders). The cohort comprised both outpatients and inpatients.

#### 2.1.3 Exclusion Criteria

Patients were excluded if they met any of the following conditions: (a) co‐infection with other pathogens (bacteria, viruses, etc.); (b) prior use of corticosteroids or antibiotics; (c) incomplete clinical or laboratory data; or (d) underlying diseases identified after screening.

#### 2.1.4 Group Assignment

After applying the criteria, 1662 eligible children were divided into pneumonia (*n* = 844) and non‐pneumonia (*n* = 818) groups. Pneumonia group: Children meeting the diagnostic criteria of the evidence‐based guidelines for diagnosis and treatment of MPP in children (2023) [8], presenting with clinical symptoms such as fever and cough, and chest imaging demonstrating pneumonia changes. Non‐pneumonia group: Children with laboratory‐confirmed MP infection who did not meet the diagnostic criteria for pneumonia, including those without typical clinical symptoms (e.g., no fever > 38.5°C lasting > 3 days, no productive cough, no tachypnea) and no radiographic evidence of pneumonia. There was no statistically significant difference in gender distribution between the two groups (*p* > 0.05), indicating comparability. Although no active follow‐up was performed, all outpatients were instructed to return immediately if symptoms worsened. Any subsequent diagnosis of pneumonia would have been recorded in the hospital's electronic medical record system.

#### 2.1.5 Ethics Statement

This study was approved by the Ethics Committee of Huaibei People's Hospital (Huaibei, Anhui, China) with the approval number 2024–097. Due to the retrospective design, the requirement for informed consent was waived by the ethics committee.

### 2.2 Reagents and Instruments

CBC parameters and whole‐blood C‐reactive protein (CRP) were measured using Mindray fully automated blood analyzers (China) or by immuno‐turbidimetric assay for serum CRP on Roche biochemical analyzers (USA) using manufacturer‐provided reagents. MP‐IgM antibody was detected by employing the MP‐IgM Antibody Detection Kit (colloidal gold method) from Weifang Kanghua Biotechnology Co. Ltd. (China). MP‐DNA was tested using the Mole 96 M Nucleic Acid Extraction and Purification System, the Yarui MA‐6000 Real‐Time Fluorescent Quantitative PCR Instrument, Shengxiang Nucleic Acid Extraction or Purification Reagents (S20015), and the MP Pathogen Nucleic Acid Detection Kit (China). Laboratory parameters were measured using standard automated analyzers (as detailed in Table S1, which provides normal reference ranges).

### 2.3 Derived Index Calculations

Calculate relevant immune‐inflammatory indices based on CBC results:

(a) NLR = NEU/LYM; (b) MLR = MON/LYM; (c) PLR = PLT/LYM; (d) PLT × LYM = PLT × LYM; (e) dNLR = NEU/(WBC‐NEU); (f) NLPR = NEU × 100/(LYM × PLT); (g) NPR = NEU × 100/PLT; (h) SII = PLT × NEU/LYM; (i) SIRI = NEU × MON/LYM; (j) AISI = PLT × NEU × MON/LYM [5, 6, 9, 10, 11].

### 2.4 Statistical Analysis

Data analysis was performed using SPSS 27.0 statistical software. Quantitative data are expressed as mean ± standard deviation (*x* ± *s*), and comparisons between two groups were conducted using the independent samples *t*‐test. Qualitative data are presented as rates (%), and intergroup comparisons were performed using the chi‐square (χ^2^) test. The diagnostic performance of each indicator was evaluated using ROC curve analysis. The area under the curve (AUC), 95% confidence interval (95% CI), sensitivity, specificity, and Youden index (Youden index = sensitivity + specificity – 1) were calculated. The optimal diagnostic cutoff value for each indicator was determined as the corresponding value where the Youden index was maximized. Multivariate logistic regression analysis was performed to identify independent predictors of pneumonia in children with MP infection. Each indicator was assigned a weight based on the absolute value of its regression coefficient to establish a simplified diagnostic scoring system. The Hosmer‐Lemeshow test was used to evaluate model fit with *p* < 0.05 indicating statistically significant differences. Age‐stratified analyses were performed across four age subgroups (≤ 3 years, 4–7 years, 8–11 years, and ≥ 12 years) using independent samples *t*‐tests to compare key indicators between the pneumonia and non‐pneumonia groups within each stratum.

### 2.5 Model Validation and Clinical Utility Assessment

To evaluate the stability and generalizability of the diagnostic model, repeated stratified 10‐fold cross‐validation was performed using Python (version 3.9.19). The process was repeated 10 times, yielding a total of 100 folds. For each fold, the model was trained on 90% of the data and tested on the remaining 10%, with stratification maintained to preserve the proportion of pneumonia cases. Performance metrics including accuracy, precision, sensitivity (recall), specificity, F1‐score, and AUC were calculated for each fold, and the mean and standard deviation were reported.

Calibration of the model was assessed using a calibration curve with 10‐fold cross‐validation, and model prediction error was quantified using the Brier score. The Brier score is calculated as:

$$\text{Brier score}=\frac{1}{N}\sum_{i=1}^{N}{\left(f_{i}-o_{i}\right)}^{2}$$

where *N* is the total number of patients, *f*_*i*_ is the predicted probability of pneumonia for the *i*‐th patient, and *o*_*i*_ is the observed outcome (1 for pneumonia, 0 for non‐pneumonia). A lower Brier score indicates better calibration. DCA was performed to evaluate the clinical net benefit of the diagnostic model across a range of high‐risk threshold probabilities. The net benefit was calculated as follows:

$$\mathrm{Net}\;\text{Benefit}=\frac{\text{True Positives}}{N}-\frac{\text{False Positives}}{N}\times \left(\frac{\text{Threshold}}{1-\text{Threshold}}\right)$$

where *N* is the total number of patients. The net benefit of the model was compared with two reference strategies: classifying all patients as pneumonia (intervention for all) and classifying all patients as non‐pneumonia (intervention for none). All statistical analyses were performed using SPSS 27.0 and Python 3.9.19 with scikit‐learn and scikit‐survival libraries.

## 3 Results

### 3.1 General Data

The pneumonia group comprised 844 cases, while the non‐pneumonia group included 818 cases. Gender distribution showed no statistically significant difference between groups (χ^2^ = 1.57, *p* = 0.210). The pneumonia group comprised 450 males (53.32%) and 394 females (46.68%), while the non‐pneumonia group included 411 males (50.24%) and 407 females (49.76%).

A significant difference existed in age distribution (*p* < 0.001). The mean age of children in the pneumonia group was (5.78 ± 3.41) years old, significantly lower than that of the non‐pneumonia group (8.62 ± 4.92) years old (*t* = 13.73, *p* < 0.001). In the pneumonia group, 27.37% (231 cases) were ≤ 3 years old, and 47.16% (398 cases) were 4–7 years old, totaling 74.53%. In contrast, the non‐pneumonia group included 19.68% (161 cases) aged 8–11 years old and 28.73% (235 cases) aged ≥ 12 years old, indicating an overall older age distribution (χ^2^ = 172.00, *p* < 0.001) (Table 1). A significant linear trend was observed: younger age was associated with a higher proportion of pneumonia cases.

**TABLE 1:** Comparison of general characteristics between pneumonia and non‐pneumonia groups.

Item	Non‐pneumonia group (*n* = 818)	Pneumonia group (*n* = 844)	*t*/χ^2^	*p*
Sex [Male/Female, *n* (%)]	411 (50.24)/407 (49.76)	450 (53.32)/394 (46.68)	χ^2^ = 1.57	0.210
Age (*x* ± *s*, years)	8.62 ± 4.92	5.78 ± 3.41	*t* = 13.73	< 0.001
Age group [*n* (%)]	—	—	χ^2^ = 172.00	< 0.001
≤ 3 years	112 (13.69)	231 (27.37)	—	—
4–7 years	310 (37.90)	398 (47.16)	—	—
8–11 years	161 (19.68)	165 (19.55)	—	—
≥ 12 years	235 (28.73)	50 (5.92)	—	—

### 3.2 Differences in Laboratory Indicators Between the Pneumonia Group and the Non‐Pneumonia Group

Comparison of laboratory indicators between the two groups revealed that the pneumonia group exhibited lower total white blood cell count (WBC), neutrophil percentage (NEUT%), absolute neutrophil count (NEUT), and absolute monocyte count (MONO) compared to the non‐pneumonia group (*p* < 0.001). Conversely, the pneumonia group demonstrated higher lymphocyte percentage (LYM%) and absolute lymphocyte count (LYM) than the non‐pneumonia group (*p* < 0.001).

Within the erythrocyte system, the pneumonia group exhibited lower red blood cell count (RBC), hemoglobin (HGB), red cell distribution width‐standard deviation (RDW‐SD), hematocrit (HCT), mean corpuscular volume (MCV), and mean corpuscular hemoglobin (MCH) compared to the non‐pneumonia group (*p* < 0.05). Among platelet‐related indicators, the pneumonia group exhibited higher total platelet count (PLT), plateletcrit (PCT), platelet distribution width (PDW), and large platelet count (P‐LCC) compared to the non‐pneumonia group (*p* < 0.001), while the large platelet ratio (P‐LCR) was lower (*p* < 0.001). Regarding CBC derived indicators, the pneumonia group exhibited lower NLR, MLR, PLR, dNLR, NLPR, NPR, SII, SIRI, and AISI compared to the non‐pneumonia group (all *p* < 0.001), while PLT × LYM was higher than the non‐pneumonia group (all *p* < 0.001) (Table 2).

**TABLE 2:** Differences in laboratory indicators between pneumonia and non‐pneumonia groups.

Item	Non‐pneumonia group (*n* = 818)	Pneumonia group (*n* = 844)	*t*	*p*
CRP (mg/L)	14.44 ± 18.60	13.02 ± 19.67	1.51	0.131
WBC (×10^9^/L)	9.91 ± 4.68	8.71 ± 3.39	6.00	< 0.001
NEUT% (%)	66.87 ± 13.70	55.68 ± 15.83	15.39	< 0.001
NEUT (×10^9^/L)	6.93 ± 4.21	5.01 ± 2.80	10.96	< 0.001
MONO% (%)	7.89 ± 2.82	7.69 ± 2.84	1.44	0.150
MONO (×10^9^/L)	0.74 ± 0.36	0.65 ± 0.30	5.73	< 0.001
LYM% (%)	24.25 ± 12.44	34.92 ± 15.18	−15.64	< 0.001
LYM (×10^9^/L)	2.16 ± 1.16	2.91 ± 1.59	−10.97	< 0.001
RBC (×10^12^/L)	4.81 ± 0.40	4.64 ± 0.42	8.46	< 0.001
HGB (g/L)	135.07 ± 12.75	128.48 ± 10.97	11.29	< 0.001
RDW‐CV (%)	12.86 ± 1.03	12.92 ± 0.91	−1.29	0.198
RDW‐SD (fL)	41.96 ± 5.31	39.74 ± 2.63	10.83	< 0.001
HCT (%)	40.48 ± 3.80	38.60 ± 3.60	10.36	< 0.001
MCV (fL)	84.14 ± 5.53	83.29 ± 4.39	3.49	< 0.001
MCH (pg)	28.14 ± 1.83	27.84 ± 2.46	2.85	0.004
MCHC (g/L)	334.50 ± 13.80	333.72 ± 19.77	0.92	0.356
PLT (×10^9^/L)	241.19 ± 72.46	305.56 ± 103.73	−14.62	< 0.001
PCT (%)	0.22 ± 0.06	0.28 ± 0.09	−15.41	< 0.001
MPV (fL)	9.36 ± 1.35	9.28 ± 1.07	1.20	0.231
PDW (fL)	15.60 ± 2.43	15.97 ± 0.71	−4.24	< 0.001
P‐LCR (%)	23.88 ± 7.65	21.76 ± 7.53	5.70	< 0.001
P‐LCC (×10^9^/L)	54.79 ± 18.40	62.77 ± 23.48	−7.69	< 0.001
NLR	4.03 ± 3.26	2.30 ± 2.18	12.81	< 0.001
MLR	0.40 ± 0.24	0.27 ± 0.18	13.23	< 0.001
PLR	141.13 ± 85.88	128.99 ± 72.53	3.12	0.002
PLT × LYM	538.92 ± 384.36	956.99 ± 837.16	−13.02	< 0.001
dNLR	2.64 ± 1.81	1.64 ± 1.31	13.04	< 0.001
NLPR	1.80 ± 1.50	0.83 ± 0.83	16.24	< 0.001
NPR	3.02 ± 1.87	1.73 ± 1.01	17.60	< 0.001
SII	981.84 ± 902.14	695.62 ± 688.49	7.29	< 0.001
SIRI	3.13 ± 3.19	1.53 ± 1.72	12.81	< 0.001
AISI	771.82 ± 885.59	479.90 ± 606.16	7.86	< 0.001

### 3.3 Logistic Regression Analysis of Pneumonia‐Related Risk Factors

Logistic regression analysis was conducted incorporating LYM%, PLT, PCT, and PLT × LYM using pneumonia status as the dependent variable. Results showed (Table 3) that LYM% (OR = 1.067, 95% CI: 1.056–1.078, Wald χ^2^ = 155.559, *p* < 0.001), PLT (OR = 1.005, 95% CI: 1.001–1.009, Wald χ^2^ = 8.285, *p* = 0.004), PCT (OR = 2961.564, 95% CI: 578.320–15068.790, Wald χ^2^ = 17.320, *p* < 0.001) were independent risk factors for pneumonia. Due to the small unit of measurement (PCT is expressed as a percentage), a one‐unit increase represents a clinically implausible change. PLT × LYM (OR = 0.999, 95% CI: 0.998–1.000, Wald χ^2^ = 16.108, *p* < 0.001) was a protective factor. Further establishing the combined diagnostic logistic regression equation: Logit (*p*) = −4.733 + 0.064 × LYM% + 0.005 × PLT + 7.993 × PCT ‐ 0.001 × PLT × LYM. The Hosmer‐Lemeshow test yielded *p* = 0.407 > 0.05, indicating good model fit.

**TABLE 3:** Regression analysis based on pneumonia and non‐pneumonia groups.

Factor	Regression coefficient	Standard error	Wald χ^2^	*p*	OR value	95% CI
LYM%	0.064	0.005	155.559	< 0.001	1.067	1.056–1.078
PLT	0.005	0.002	8.285	0.004	1.005	1.001–1.009
PCT	7.993	1.921	17.320	< 0.001	2961.564	578.320–15068.790
PLT × LYM	−0.001	0.001	16.108	< 0.001	0.999	0.998–1.000
Constant	−4.733	0.304	241.557	< 0.001	0.009	0.005–0.016

### 3.4 Diagnostic Performance Analysis of Indicators Between Pneumonia and Non‐Pneumonia Groups

**FIGURE 1:**
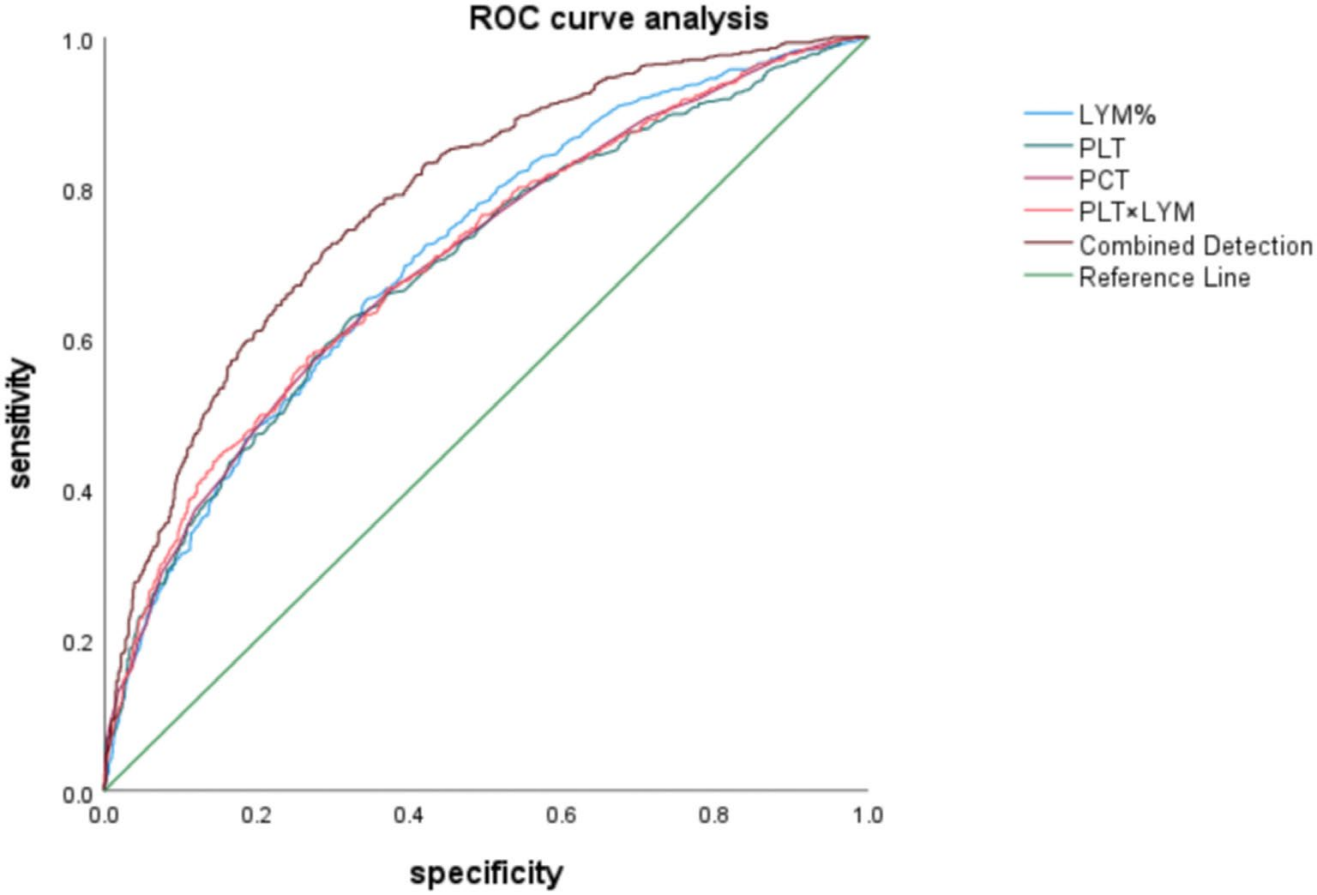
Diagnostic performance of indicators in pneumonia and non‐pneumonia groups from ROC curve analysis.

ROC curve analysis revealed (Table 4, Figure 1) that among single indicators, PLT × LYM demonstrated optimal diagnostic performance with an AUC of 0.706 (95% CI: 0.681–0.730), sensitivity of 0.577, specificity of 0.732, and Youden index of 0.309, followed by LYM% (AUC = 0.710, 95% CI: 0.686–0.735, Youden index 0.308), PLT (AUC = 0.696, 95% CI: 0.671–0.721, Youden index 0.304), PCT (AUC = 0.702, 95% CI: 0.677–0.727, Youden index 0.296). The combined detection yielded an AUC of 0.784 (95% CI: 0.763–0.806), sensitivity of 0.717, specificity of 0.713, and a Youden index of 0.430, demonstrating superior diagnostic performance compared to individual indicators.

**TABLE 4:** Diagnostic performance of indicators in pneumonia and non‐pneumonia groups.

Factor	Cutoff value	AUC	95% CI	Sensitivity	Specificity	Youden index
LYM%	26.850	0.710	0.686–0.735	0.653	0.655	0.308
PLT	260.500	0.696	0.671–0.721	0.628	0.676	0.304
PCT	0.255	0.702	0.677–0.727	0.569	0.727	0.296
PLT × LYM	643.600	0.706	0.681–0.730	0.577	0.732	0.309
Combined detection	0.487	0.784	0.763–0.806	0.717	0.713	0.430

### 3.5 Development of a Simplified Diagnostic Scoring System and Its Clinical Application Combined With Logit(P)

**FIGURE 2:**
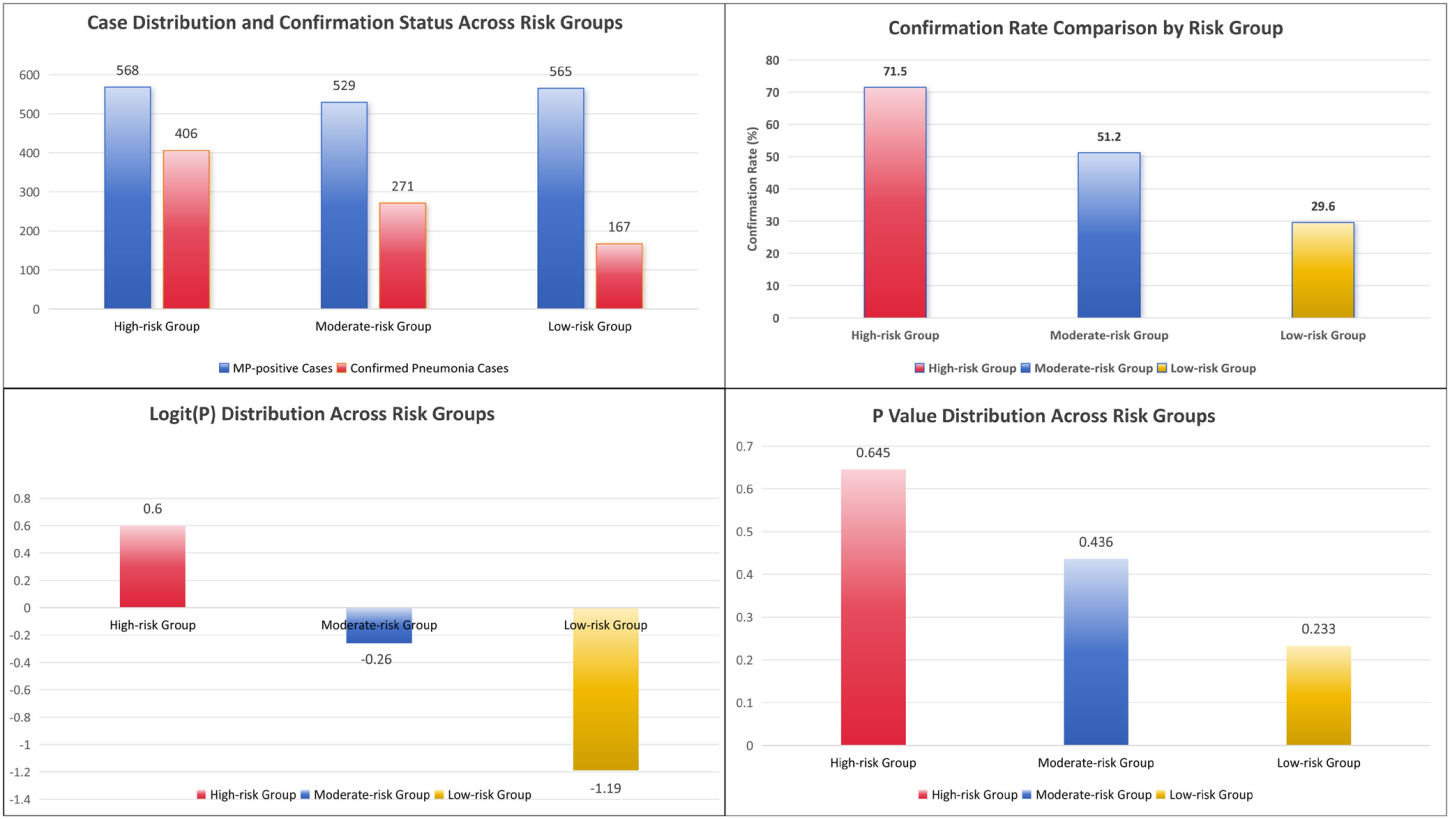
Comparison of pneumonia risk stratification analysis results.

The scoring weights were determined based on the absolute values of coefficients for each indicator in the logistic regression equation. Combined with the optimal cutoff value from the ROC curve, a scoring standard was established to construct a simplified diagnostic scoring table (Table 5). This scoring table ranges from 0 to 8 points. By substituting different scoring combinations, the corresponding Logit (*p*) and disease probability (*p*‐value) were calculated to establish risk stratification criteria. The high‐risk group (≥ 6 points) corresponds to Logit (*p*) values between −2.71 and 4.73 and *p* values between 0.062 and 0.991, comprising 568 cases. Among these, 406 were diagnosed with pneumonia, yielding a diagnostic accuracy of 71.5%. The moderate‐risk group (3–5 points) corresponds to Logit (*p*) values between −2.07 and 2.48, with *p* values ranging from 0.112 to 0.923, comprising 529 cases. Among these, 271 were confirmed with pneumonia, yielding a confirmation rate of 51.2%. The low‐risk group (≤ 2 points) corresponded to Logit (*p*) values between −3.13 and 2.39, with *p* values ranging from 0.042 to 0.916. This group comprised 565 cases, of which 167 were diagnosed with pneumonia, yielding a diagnosis rate of 29.6% (Table 6, Figure 2).

**TABLE 5:** Simplified diagnostic scoring system (total score 0–8).

Diagnostic indicator	Criteria	Score	Efficacy basis
PLT × LYM	≥ 643.600	3	Youden Index = 0.309, AUC = 0.706
LYM% (%)	> 26.850	2	Youden Index = 0.308, AUC = 0.710
PLT (×10^9^/L)	> 260.500	2	Youden Index = 0.304, AUC = 0.696
PCT	> 0.255	1	Youden Index = 0.296, AUC = 0.702

**TABLE 6:** Results of pneumonia risk stratification analysis.

Risk group	Score range	Total cases (*n*)	Confirmed cases (*n*)	Non‐confirmed cases (*n*)	Confirmation rate (%)	Logit (*p*) range	*p* range
High‐risk group	≥ 6	568	406	162	71.5	−2.71–4.73	0.062–0.991
Moderate‐risk group	3–5	529	271	258	51.2	−2.07–2.48	0.112–0.923
Low‐risk group	0–2	565	167	398	29.6	−3.13–2.39	0.042–0.916

### 3.6 Age‐Stratified Analysis of Key Indicators

To evaluate whether the observed differences in immune‐inflammatory indicators were confounded by age, we performed stratified analyses across four age subgroups: ≤ 3 years, 4–7 years, 8–11 years, and ≥ 12 years. As shown in Table 7, within each age stratum, the pneumonia group exhibited significantly higher levels of LYM%, PLT, PCT, and PLT × LYM compared with the non‐pneumonia group (all *p* < 0.001). These results indicate that the associations between these indicators and pneumonia are not merely attributable to age differences, but reflect pathological changes associated with MPP.

**TABLE 7:** Comparison of laboratory indicators between the pneumonia and non‐pneumonia groups across age strata.

Item	Age group	Non‐pneumonia group (*n*(*x* ± *s*))	Pneumonia group (*n*(*x* ± *s*))	*t*	*p*
LYM%	≤ 3岁	112 (30.15 ± 14.65)	231 (42.13 ± 16.51)	6.81	< 0.001
	4–7岁	310 (25.32 ± 12.85)	398 (33.92 ± 14.01)	8.49	< 0.001
	8–11岁	161 (22.14 ± 11.09)	165 (29.38 ± 13.03)	5.41	< 0.001
	≥ 12岁	235 (21.48 ± 10.37)	50 (27.86 ± 10.88)	3.79	< 0.001
PLT	≤ 3岁	112 (246.13 ± 71.09)	231 (313.33 ± 109.16)	6.83	< 0.001
	4–7岁	310 (240.20 ± 74.55)	398 (302.55 ± 105.46)	9.21	< 0.001
	8–11岁	161 (240.01 ± 72.67)	165 (298.98 ± 92.63)	6.40	< 0.001
	≥ 12岁	235 (240.94 ± 70.51)	50 (315.30 ± 98.73)	5.06	< 0.001
PCT	≤ 3岁	112 (0.23 ± 0.06)	231 (0.28 ± 0.09)	6.48	< 0.001
	4–7岁	310 (0.23 ± 0.06)	398 (0.28 ± 0.09)	9.42	< 0.001
	8–11岁	161 (0.23 ± 0.06)	165 (0.28 ± 0.08)	5.94	< 0.001
	≥ 12岁	235 (0.21 ± 0.07)	50 (0.30 ± 0.08)	7.56	< 0.001
PLT × LYM	≤ 3岁	112 (687.21 ± 453.97)	231 (1258.84 ± 1000.65)	7.28	< 0.001
	4–7岁	310 (584.37 ± 428.16)	398 (893.47 ± 830.85)	6.41	< 0.001
	8–11岁	161 (520.31 ± 342.26)	165 (764.77 ± 527.07)	4.98	< 0.001
	≥ 12岁	235 (421.02 ± 261.56)	50 (702.33 ± 426.40)	4.49	< 0.001

### 3.7 Internal Validation and Model Stability

**FIGURE 3:**
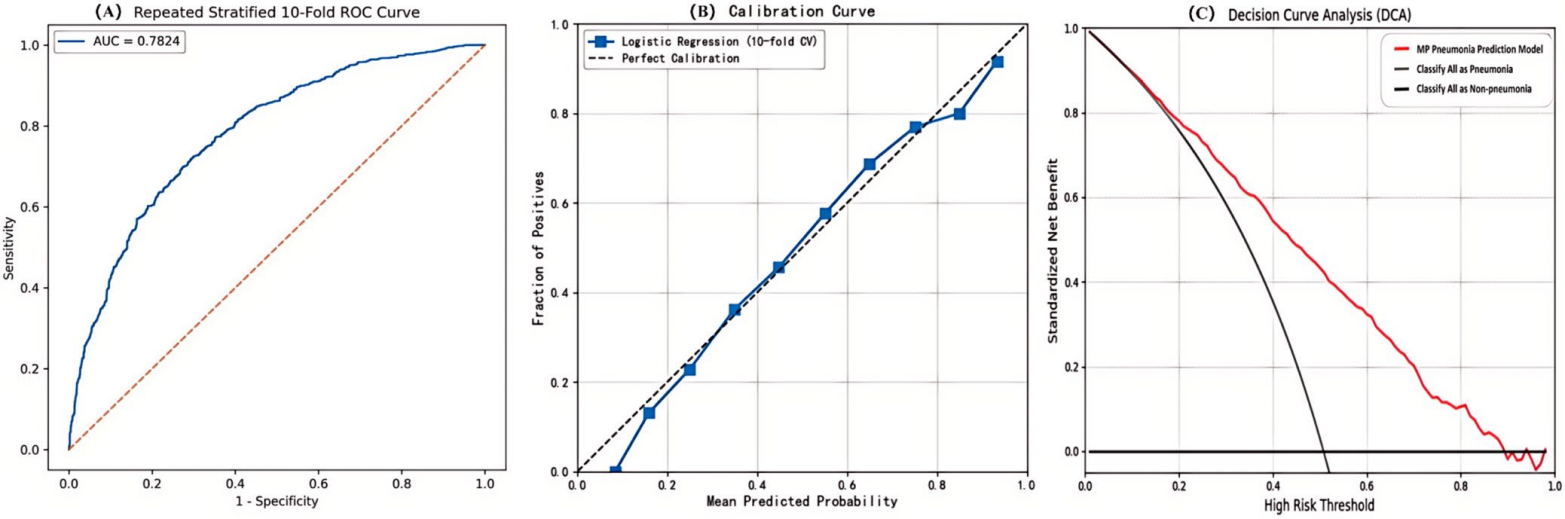
Internal validation of the diagnostic model. (A) ROC curves from repeated stratified 10‐fold cross‐validation (10 repeats, total 100 folds). The mean AUC was 0.7824 ± 0.0353. (B) Calibration curve with Brier score of 0.1918 ± 0.0164. (C) Decision curve analysis showing that the model provides superior net benefit compared with the “Classify All as Pneumonia” and “Classify All as Non‐pneumonia” strategies across a wide range of clinically relevant thresholds.

To assess the stability and generalizability of the diagnostic model, repeated stratified 10‐fold cross‐validation was performed (Figure 3A, 10 repeats, with a total of 100 folds). The model demonstrated consistent performance across all folds, with a mean AUC of 0.7824 ± 0.0353, mean accuracy of 0.7111 ± 0.0346, mean sensitivity of 0.6970 ± 0.0510, and mean specificity of 0.7256 ± 0.0495. The calibration curve showed good agreement between predicted probabilities and observed outcomes, with a Brier score of 0.1918 ± 0.0164 (Figure 3B). DCA further confirmed that the model provided a positive net benefit over a wide range of risk thresholds compared with classifying all patients as pneumonia or as non‐pneumonia (Figure 3C), indicating its potential value in guiding clinical decisions.

## 4 Discussion

MP, a type of cell‐wall‐less prokaryotic microorganism, is primarily transmitted through respiratory droplets. Following infection, the incubation period lasts approximately 2–3 weeks, making it prone to causing cluster infections in crowded settings such as schools and childcare facilities. Clinical manifestations of MP infection in children are age‐related: infants and toddlers under 5 years old typically present with mild or subclinical symptoms, while school‐aged children and adolescents are more likely to develop typical pneumonia. MP infection is a major cause of respiratory infections in children. The MPP often presents atypically, making early diagnosis challenging [12]. Particularly concerning is the recurring peak of MP infections in 2023, coupled with the emerging epidemiological feature of macrolide antibiotic resistance, which increases the likelihood of severe disease progression in infected children [13]. Consequently, early diagnosis and differential diagnosis are critically important.

Laboratory diagnosis of MP lacks specificity. While pathogen detection serves as the definitive diagnostic basis, it has limitations. Current diagnostic methods include pathogen detection and serological testing, each with distinct advantages and drawbacks. For instance, MP‐IgM antibodies may not be detectable in the early stages of infection, potentially leading to false‐negative results. Nucleic acid testing, however, is costly and difficult to implement widely in primary care settings [14]. CBC, as a routine clinical test, offers the advantages of speed, convenience, and low cost. Its derived immune‐inflammatory indicators can reflect real‐time inflammatory responses and immune status, and have been widely applied in the diagnosis of various infectious diseases [15]. Studies confirm that children with MP infection complicated by pneumonia exhibit significant immune‐inflammatory responses particularly in the early disease stage. These manifestations include platelet activation and enhanced lymphocyte function. Changes in these indicators closely correlate with disease severity, thus providing critical biological references for early diagnosis [16, 17].

This study conducted a multi‐dimensional analysis of clinical data and laboratory indicators in pediatric MP infection patients with and without pneumonia. It clarified the age distribution characteristics of childhood pneumonia onset and identified key laboratory diagnostic markers, providing important reference for early and precise clinical diagnosis. Findings revealed a significant age‐related pattern in childhood pneumonia incidence, with children under 7 years old constituting the high‐risk group (74.53%). This age distribution may stem from the immature immune systems and weaker pulmonary defense mechanisms in younger children, making them more susceptible to pathogen invasion. This finding offers direction for targeted preventive interventions in high‐risk populations. Gender showed no significant influence on pneumonia incidence, with no statistically significant differences in gender distribution between groups. Laboratory indicators in the pneumonia group exhibited characteristic alterations, showing statistically significant differences compared to the non‐pneumonia group (*p* < 0.05): White blood cell system: WBC, NEUT%, NEUT, and MONO were significantly decreased, while LYM% and LYM were significantly increased. Red blood cell system: RBC, HGB, RDW‐SD, HCT, MCV, and MCH all showed a decreasing trend. Platelet indicators: PLT, PCT, PDW, and P‐LCC were significantly elevated, while P‐LCR was significantly decreased. CBC derived immune‐inflammatory indicators: NLR, MLR, PLR, dNLR, NLPR, NPR, SII, SIRI, and AISI were all significantly decreased, whereas PLT × LYM was significantly increased. These indicators provide objective references for the clinical differentiation of pneumonia.

LYM% and LYM levels in the pneumonia group were significantly higher than those in the non‐pneumonia group. LYM% emerged as an independent diagnostic risk factor (OR = 1.067, 95% CI: 1.056–1.078), with an ROC curve AUC of 0.710. This finding aligns closely with the adaptive immune response mechanism triggered by MP infection [18]. As an intracellular pathogen, MP invades the body by activating the Toll‐like receptor (TLR) pathway, stimulating lymphocyte proliferation and activation, and enhancing anti‐pathogen immunity, which leads to a characteristic increase in peripheral blood lymphocyte counts [19]. As core effector cells of adaptive immunity, changes in lymphocyte percentage directly reflect the body's anti‐infective immune status [20].

The PLT level in the pneumonia group was also significantly higher than that in the non‐pneumonia group and served as an independent diagnostic risk factor (OR = 1.005, 95% CI: 1.001–1.009), confirming the key role of platelets in the inflammatory response to MP infection [21]. Platelets not only participate in hemostasis and coagulation but also regulate the development of inflammation by releasing inflammatory mediators such as platelet‐activating factor (PAF) and interleukins. Elevated platelet counts and increased mean platelet volume indicate enhanced platelet activation, correlating with the severity of MP‐induced inflammatory responses [22]. MP infection‐induced pulmonary inflammation stimulates bone marrow megakaryocyte proliferation, leading to increased platelet production. Elevated platelet counts directly reflect the degree of platelet activation and correlate positively with the extent of pulmonary inflammatory infiltration [9, 23].

PLT × LYM, a composite indicator derived from LYM and PLT, demonstrated optimal diagnostic performance in this study (AUC = 0.706, 95% CI: 0.681–0.730, Youden index = 0.309) and served as a protective factor (OR = 0.999, 95% CI: 0.998–1.000). A significant increase was observed in the pneumonia group relative to the non‐pneumonia group. This characteristic change stems from enhanced synergistic immune function between lymphocytes and platelets during MP infection complicated by pneumonia [24]. After invading the body, MP can stimulate macrophages to release inflammatory cytokines, activate lymphocytes and platelets, leading to lymphocyte proliferation and platelet activation/aggregation, while simultaneously inhibiting neutrophil release into peripheral blood, resulting in decreased neutrophil counts [19]. Furthermore, lower respiratory tract infections in pneumonia patients primarily occur in the alveoli and pulmonary interstitium, where intense and sustained immune responses frequently take place. This leads to the recruitment of large numbers of immune cells, such as neutrophils and macrophages, to the local lung tissue, forming significant inflammatory infiltrates. This process aids in protective immunity but reduces their release into the peripheral blood. Thus, peripheral blood leukocyte counts often remain unremarkably elevated or even persist within normal ranges or show only mild increases due to cellular migration into tissues [25].

MP infection can also trigger excessive inflammatory responses, leading to cytokine storms that precipitate complications such as pulmonary consolidation and pleural effusion [26]. Abnormal changes in indicators like PLT × LYM and LYM% reflect this inflammatory state. The greater the degree of abnormality, the more intense the inflammatory response and the more severe the condition [27]. Concurrently, MP‐induced pulmonary inflammation can compromise the respiratory mucosal barrier, increasing the risk of co‐infection with other pathogens and further exacerbating inflammatory responses and immune dysregulation [28]. As a protective factor, elevated PLT × LYM levels indicated that the underlying mechanism involved a synergistic enhancement of both lymphocyte and platelet functions. This also signifies the enhancement of pathogen clearance capacity against MP, reducing pulmonary inflammatory damage and lowering the risk of pneumonia progression to severe disease. Clinically, this can serve as a prognostic indicator, guiding more aggressive interventions for patients with low expression.

The simplified scoring system developed in this study, based on multivariate logistic regression coefficients, provided a quantitative basis for clinical diagnosis, disease assessment, and treatment decision‐making through its three‐tier risk stratification framework. The high‐risk group (≥ 6 points) demonstrated a pneumonia confirmation rate of 71.5%, with Logit (*p*) ranging from −2.71 to 4.73 and disease probability from 0.062 to 0.991. These patients predominantly exhibited immune‐inflammatory features characterized by PLT × LYM, LYM%, PLT, and PCT, indicating severe pulmonary inflammatory infiltration. Immediate targeted treatment with macrolide antibiotics is required, prioritizing chest imaging to assess lesion extent and closely monitoring for complications such as pulmonary consolidation and pleural effusion. Immunomodulatory therapy should be considered when necessary [29, 30]. The pneumonia confirmation rate in the moderate‐risk group (3–5 points) was 51.2%, with Logit (*p*) ranging from −2.07 to 2.48 and with disease probability ranging from 0.112 to 0.923. Immunological and inflammatory indicators show relatively mild abnormalities, indicating that some patients are in the early stages of infection or have mild disease. Dynamic monitoring of CBC and derived indicators over 24–48 h is required. Treatment plans should be adjusted based on changes in the score or fluctuations in core indicators, avoiding overtreatment [6, 27]. Low‐risk group (≤ 2 points): Pneumonia confirmation rate only 29.6%, Logit (*p*) −3.13 to 2.39, disease probability 0.042 to 0.916. Most cases represent early‐stage or mild MP infections with mild immune‐inflammatory responses and low‐risk of pulmonary involvement. Clinical management should focus on symptomatic treatment without immediate antimicrobial use. The family should be informed to closely monitor symptoms and promptly seek follow‐up care if a persistent high fever or worsening cough occurs. This approach reduces unnecessary antimicrobial use and lowers the risk of macrolide antibiotic resistance [31, 32]. The core advantage of the scoring system lies in transforming complex statistical models into intuitive quantitative tools. It enables rapid application without specialized statistical knowledge, effectively addressing diagnostic limitations in primary healthcare settings [33]. Compared to traditional pathogen detection, this scoring system relies on routine CBC indicators, offering low detection costs and completing scoring within approximately 30 min from sample collection. It meets the core clinical need for rapid early diagnosis, demonstrating the significant value of low‐cost biomarkers in primary care settings.

Furthermore, internal validation using repeated stratified 10‐fold cross‐validation demonstrated good model stability, with a mean AUC of 0.7824 ± 0.0353 and consistent performance across folds. The calibration curve demonstrated a favorable Brier score of 0.1918 ± 0.0164, and DCA confirmed positive net benefit across a wide range of clinically relevant thresholds. These findings support the model's robustness and clinical utility, particularly in primary care settings with limited diagnostic resources.

The study has several limitations. First, although patients with immunodeficiency were excluded, undiagnosed or subclinical immune deficiencies cannot be completely ruled out, which may have introduced residual confounding. Future prospective studies incorporating immunological profiling would help clarify this issue. Second, a significant age difference existed between the two groups. Nevertheless, age‐stratified analysis confirmed that key indicators remained significantly higher in the pneumonia group across all age strata, and internal validation via repeated 10‐fold cross‐validation (mean AUC = 0.7824 ± 0.0353) supported model stability. Finally, this was a single‐center study, and external validation in multicenter cohorts is needed to confirm generalizability.

In summary, this study demonstrates that CBC‐derived immune‐inflammatory indicators, particularly PLT × LYM, LYM%, PLT, and PCT, have significant diagnostic value for MPP in children. The novelty of this study lies in the development of a simple, low‐cost scoring system that translates complex statistical models into an easy‐to‐use clinical tool. The model's robustness is supported by repeated stratified 10‐fold cross‐validation (mean AUC = 0.7824 ± 0.0353), good calibration (Brier score = 0.1918 ± 0.0164), and positive net benefit on DCA. This scoring system enables rapid risk stratification without specialized equipment or expertise, making it particularly suitable for primary healthcare settings where advanced diagnostic resources are limited. Its use may facilitate early diagnosis, reduce unnecessary antibiotic prescriptions, and help mitigate the growing challenge of macrolide resistance. Future multicenter studies with external validation are warranted to further establish its clinical utility.

## Funding

This work was supported by Natural Science Key Project of Bengbu Medical University, 2024byzd324.

## Conflicts of Interest

The authors declare no conflicts of interest.

## Supporting information


**Table S1:** Normal reference ranges for laboratory parameters.

## Data Availability

The data that support the findings of this study are not publicly available due to patient privacy and ethical restrictions imposed by the Ethics Committee of Huaibei People's Hospital (approval number 2024–097). De‐identified data may be requested from the corresponding author (Ling Ge, gelinghbsrmyy@163.com) upon reasonable request, subject to institutional ethical approval.
