# Supplementary material for: Utility of a Combined Diagnostic and Severity Scoring System Based on Complete Blood Count and Derived Immune‐Inflammatory Indicators in Children With Mycoplasma pneumoniae Pneumonia
Source: J Clin Lab Anal. 2026 Jun 6;40(13):e70263. doi: 10.1002/jcla.70263 (PMC13371284; doi:10.1002/jcla.70263)
Supplement: Supplementary file 1 — Table S1: Normal reference ranges for laboratory parameters. [file JCLA-40-e70263-s001.docx]

**TABLE S1** Normal reference ranges for laboratory parameters

| **Parameter** | **Unit** | **Age Group** | **Reference Range** | **References** |
| --- | --- | --- | --- | --- |
| **CRP** | **mg/L** | Children | < 5.0 | 1,2 |
| **WBC** | **×10⁹/L** | 28 days-6 months | 4.3～14.2 | 2 |
|  |  | 6 months-1 year | 4.8～14.6 | 2 |
|  |  | 1-2 years | 5.1～14.1 | 2 |
|  |  | 2-6 years | 4.4～11.9 | 2 |
|  |  | 6-13 years | 4.3～11.3 | 2 |
|  |  | 13-18 years | 4.1～11.0 | 2 |
| **NEUT%** | **%** | 28 days-6 months | 7~56 | 2 |
|  |  | 6 months-1 year | 9~57 | 2 |
|  |  | 1-2 years | 13~55 | 2 |
|  |  | 2-6 years | 22~65 | 2 |
|  |  | 6-13 years | 31~70 | 2 |
|  |  | 13-18 years | 37~77 | 2 |
| **NEUT** | **×10⁹/L** | 28 days-6 months | 0.6~7.5 | 2 |
|  |  | 6 months-1 year | 0.8~6.4 | 2 |
|  |  | 1-2 years | 0.8~5.8 | 2 |
|  |  | 2-6 years | 1.2~7.0 | 2 |
|  |  | 6-13 years | 1.6~7.8 | 2 |
|  |  | 13-18 years | 1.8~8.3 | 2 |
| **MONO%** | **%** | 28 days-6 months | 3~16 | 2 |
|  |  | 6 months-2 years | 2~13 | 2 |
|  |  | 2-18 years | 2~11 | 2 |
| **MONO** | **×10⁹/L** | 28 days-6 months | 0.15~1.56 | 2 |
|  |  | 6 months-1 year | 0.17~1.06 | 2 |
|  |  | 1-2 years | 0.18~1.13 | 2 |
|  |  | 2-6 years | 0.12~0.93 | 2 |
|  |  | 6-13 years | 0.13~0.76 | 2 |
|  |  | 13-18 years | 0.14~0.74 | 2 |
| **LYM%** | **%** | 28 days-6 months | 26~83 | 2 |
|  |  | 6 months-1 year | 31~81 | 2 |
|  |  | 1-2 years | 33~77 | 2 |
|  |  | 2-6 years | 23~69 | 2 |
|  |  | 6-13 years | 23~59 | 2 |
|  |  | 13-18 years | 17~54 | 2 |
| **LYM** | **×10⁹/L** | 28 days-6 months | 2.4~9.5 | 2 |
|  |  | 6 months-1 year | 2.5~9.0 | 2 |
|  |  | 1-2 years | 2.4~8.7 | 2 |
|  |  | 2-6 years | 1.8~6.3 | 2 |
|  |  | 6-13 years | 1.5~4.6 | 2 |
|  |  | 13-18 years | 1.2~3.8 | 2 |
| **RBC** | **×10^12^/L** | 28 days-6 months | 3.3～5.2 | 2 |
|  |  | 6 months-6 years | 4.0～5.5 | 2 |
|  |  | 6-13 years | 4.2～5.7 | 2 |
|  |  | 13-18 years(male) | 4.5～5.9 | 2 |
|  |  | 13-18 years(female) | 4.1～5.3 | 2 |
| **HGB** | **g/L** | 28 days-6 months | 97~183 | 2 |
|  |  | 6 months-1 year | 97~141 | 2 |
|  |  | 1-2 years | 107~141 | 2 |
|  |  | 2-6 years | 112~149 | 2 |
|  |  | 6-13 years | 118~156 | 2 |
|  |  | 13-18 years(male) | 129~172 | 2 |
|  |  | 13-18 years(female) | 114~154 | 2 |
| **RDW-CV** | **%** | 28 days-2 years | 11.8～16.0 | 3 |
|  |  | 2-18 years | 11.9～14.5 | 3,4 |
| **RDW-SD** | **fL** | 28 days-2 years | 36.1～51.3 | 3 |
|  |  | 2-18 years | 34.8～43.3 | 3 |
| **HCT** | **%** | 28 days-6 months | 28~52 | 2 |
|  |  | 6 months-1 year | 30~41 | 2 |
|  |  | 1-2 years | 32~42 | 2 |
|  |  | 2-6 years | 34~43 | 2 |
|  |  | 6-13 years | 36~46 | 2 |
|  |  | 13-18 years(male) | 39~51 | 2 |
|  |  | 13-18 years(female) | 36~47 | 2 |
| **MCV** | **fL** | 28 days-6 months | 73~104 | 2 |
|  |  | 6 months-2 years | 72~86 | 2 |
|  |  | 2-6 years | 76~88 | 2 |
|  |  | 6-13 years | 77~92 | 2 |
|  |  | 13-18 years | 80~100 | 2 |
| **MCH** | **pg** | 28 days-6 months | 24~37 | 2 |
|  |  | 6 months-6 years | 24~30 | 2 |
|  |  | 6-18 years | 25~34 | 2 |
| **MCHC** | **g/L** | 28 days-6 months | 309~363 | 2 |
|  |  | 6 months-18 years | 310~355 | 2 |
| **PLT** | **×10⁹/L** | 28 days-6 months | 183~614 | 2 |
|  |  | 6 months-1 year | 190~579 | 2 |
|  |  | 1-2 years | 190~524 | 2 |
|  |  | 2-6 years | 188~472 | 2 |
|  |  | 6-12 years | 167~453 | 2 |
|  |  | 12-18 years | 150~407 | 2 |
| **PCT** | **%** | 28 days-2 years | 0.18～0.48 | 3 |
|  |  | 2-18 years | 0.17～0.43 | 3 |
| **MPV** | **fL** | 28 days-2 years(male) | 7.9～12.1 | 3 |
|  |  | 28 days-2 years(female) | 7.2～12.0 | 3 |
|  |  | 2-18 years | 8.3～12.8 | 3 |
| **PDW** | **fL** | 28 days-2 years | 9.6～16.5 | 3 |
|  |  | 2-18 years | 9.0～17.6 | 3,4 |
| **P-LCR** | **%** | 28 days-2 years | 11.1～39.7 | 3 |
|  |  | 2-18 years | 12.7～48.3 | 3 |
| **P-LCC** | **×10⁹/L** | 28 days-18 years | 30~90 | 3 |

**Note:** For CBC-derived immune-inflammatory indicators (NLR, MLR, PLR, PLT×LYM, dNLR, NLPR, NPR, SII, SIRI, and AISI), no established pediatric reference ranges are available in the literature, as these are calculated indices.The primary conclusions of this study are based on comparisons between groups rather than on individual classification relative to reference ranges.

**REFERENCES**

1. Dai SZ, Song MY, Mi LL, et al. Percentile distribution curves and reference values of C-reactive protein in children in Beijing based on the GAMLSS model [in Chinese]. *Laboratory Medicine*. 2023;38(10):977-979.

2. National Health Commission of the People’s Republic of China. Reference intervals of blood cell analysis for children (WS/T 779-2021) [in Chinese]. *Beijing: Standards Press of China*; 2021.

3. Huang LS, Li G, Zhang C. Reference interval investigation of venous blood routine in children aged 0-12 years in Central China [in Chinese]. *Laboratory Medicine*. 2017;32(7):610-613.

4. Xi Y, Bai Y. Diagnostic value of red blood cell distribution width, platelet distribution width, and red blood cell distribution width to platelet ratio in children with hemophagocytic lymphohistiocytosis. *J Clin Lab Anal*. 2021;35(9):e23909.
